# Occipital hypometabolism is a risk factor for conversion to Parkinson’s disease in isolated REM sleep behaviour disorder

**DOI:** 10.1007/s00259-023-06289-y

**Published:** 2023-06-13

**Authors:** Giulia Carli, Sanne K. Meles, Annette Janzen, Elisabeth Sittig, Rosalie V. Kogan, Daniela Perani, Wolfgang H. Oertel, Klaus L. Leenders, the REMPET Working Group

**Affiliations:** 1 Department of Nuclear Medicine and Molecular Imaging, University of Groningen, University Medical Center Groningen, Groningen, the Netherlands; 2 Department of Neurology, University of Groningen, University Medical Center Groningen, Groningen, the Netherlands; 3 Department of Neurology, Philipps-Universität Marburg, Marburg, Germany; 4 Department of Internal Medicine, Sierra View Medical Center, Porterville, CA USA; 5 School of Psychology, Vita-Salute San Raffaele University, Milan, Italy; 6 In Vivo Human Molecular and Structural Neuroimaging Unit, Division of Neuroscience, IRCCS San Raffaele Scientific Institute, Milan, Italy; 7 Institute for Neurogenomics, Helmholtz Center for Health and Environment, Munich, Germany

**Keywords:** Isolated REM sleep behaviour disorder, Short-term conversion, ^18^F-FDG PET, Occipital hypometabolism

## Abstract

**Purpose:**

Isolated REM sleep behaviour disorder (iRBD) patients are at high risk of developing clinical syndromes of the α-synuclein spectrum. Progression markers are needed to determine the neurodegenerative changes and to predict their conversion. Brain imaging with ^18^F-FDG PET in iRBD is promising, but longitudinal studies are scarce. We investigated the regional brain changes in iRBD over time, related to phenoconversion.

**Methods:**

Twenty iRBD patients underwent two consecutive ^18^F-FDG PET brain scans and clinical assessments (3.7 ± 0.6 years apart). Seventeen patients also underwent ^123^I-MIBG and ^123^I-FP-CIT SPECT scans at baseline. Four subjects phenoconverted to Parkinson’s disease (PD) during follow-up. ^18^F-FDG PET scans were compared to controls with a voxel-wise single-subject procedure. The relationship between regional brain changes in metabolism and PD-related pattern scores (PDRP) was investigated.

**Results:**

Individual hypometabolism t-maps revealed three scenarios: (1) normal ^18^F-FDG PET scans at baseline and follow-up (*N* = 10); (2) normal scans at baseline but occipital or occipito-parietal hypometabolism at follow-up (*N* = 4); (3) occipital hypometabolism at baseline and follow-up (*N* = 6). All patients in the last group had pathological ^123^I-MIBG and ^123^I-FP-CIT SPECT. iRBD converters (*N* = 4) showed occipital hypometabolism at baseline (third scenario). At the group level, hypometabolism in the frontal and occipito-parietal regions and hypermetabolism in the cerebellum and limbic regions were progressive over time. PDRP *z*-scores increased over time (0.54 ± 0.36 per year). PDRP expression was driven by occipital hypometabolism and cerebellar hypermetabolism.

**Conclusions:**

Our results suggest that occipital hypometabolism at baseline in iRBD implies a short-term conversion to PD. This might help in stratification strategies for disease-modifying trials.

**Supplementary Information:**

The online version contains supplementary material available at 10.1007/s00259-023-06289-y.

## Introduction

Isolated REM sleep behaviour disorder (iRBD) patients are at high risk of developing clinical syndromes of the α-synuclein spectrum [1]. Most iRBD patients (> 90%) will develop Parkinson’s disease (PD), dementia with Lewy bodies (DLB), or (rarely) multiple system atrophy (MSA) within 5–20 years after diagnosis [2]. iRBD patients are ideal candidates for potential disease-modifying trials [3]. However, the progression and clinical trajectory rates are heterogeneous [4], making it difficult to predict which iRBD patient will develop a particular condition and when. Longitudinal neuroimaging studies may be of use in making such predictions.

^18^F-2-Fluoro-2-deoxy-d-glucose positron emission tomography (^18^F-FDG PET) allows assessment of cerebral glucose metabolism and is used in the workup of parkinsonism [5] and dementia [6]. Using single-subject statistical parametric mapping (SPM) t-maps, expert readers can identify typical patterns of hypometabolism in individual cases [7]. This semi-quantitative approach can be used to distinguish between PD and MSA [8] and to predict the development of dementia in PD patients [9]. Quantitative methods include univariate voxel-wise or region of interest (ROI)–based analyses. Glucose metabolism changes have also been investigated in several neurodegenerative disorders with spatial covariance analysis, which provides disease-related patterns that disclose the network-level brain changes in specific conditions [10]. Such patterns are applied case-by-case to calculate to which degree an individual’s scan is compatible with the disease pattern.

Several ^18^F-FDG PET studies have been performed in iRBD ([11] for review). However, most of these studies are cross-sectional and based on group analysis, and the results are conflicting. Longitudinal neuroimaging studies are scarce. Kim et al. [12] applied spatial covariance and regional analyses to scans of 20 iRBD patients at baseline and after 2 and 4 years. They found that metabolism in the putamen increased over time, and that metabolism in the motor cortices and frontal cortex decreased. These changes were related to conversion to PD/DLB. A study of brain perfusion in 37 iRBD patients scanned at baseline and after 1 year found that regional hypoperfusion in the frontal cortex normalised over time, perhaps suggesting early compensatory mechanisms [13]. Contradictory to cross-sectional studies [11] and two older longitudinal perfusion studies [14, 15], Baril et al. [13] and Kim et al. [12] did not find the expected increases in the hippocampus or decreases in the occipital cortex over time.

Spatial covariance analysis has also been applied to ^18^F-FDG PET in iRBD by us and other authors [12, 16–21]. We previously investigated the expression of a PD-related network (PDRP) in a cohort of 20 iRBD patients scanned twice, approximately 4 years apart [21]. This was characterised by a relatively increased activity in the cerebellum, pons, thalamus, putamen/globus pallidus, and motor cortex and moderately decreased metabolism in the lateral premotor and parieto-occipital cortex. We showed that PDRP expression increased from baseline to follow-up and that a greater rate-of-change of PDRP expression is associated with conversion to PD. However, in that study, the contribution of single brain regions to PDRP expression was not addressed. The abovementioned inconsistencies warrant further evaluation of regional longitudinal brain metabolic changes. So far, no study has evaluated the topography of hypometabolism and hypermetabolism patterns over time in iRBD patients at the individual level.

This study evaluates the longitudinal regional brain changes in iRBD with ^18^F-FDG PET. Although most iRBD patients share a final common clinical syndrome, their trajectories towards this common phenotype may differ. Therefore, it is useful to investigate regional changes on a case-by-case basis instead of at the group level. We hypothesised that this heterogeneity would be reflected in their individual brain hypometabolism and hypermetabolism maps. Specifically, we expected different topographical changes over time in iRBD patients who converted to PD at follow-up compared to those who did not. To this end, we applied an optimised statistical parametric mapping (SPM) procedure [7] to obtain single-subject hypo/hypermetabolism maps at baseline and follow-up (approximately 4 years later), which were visually rated by experts. In addition, we investigated the progression of regional hypometabolism without an a priori hypothesis using two data-driven univariate approaches (voxel-based and ROI analyses). We also evaluated the contribution of regional changes to PDRP expression over time and their relationship to clinical follow-up measures. Furthermore, cardiac noradrenergic sympathetic and presynaptic dopaminergic nigrostriatal innervations were assessed in 17 out of 20 subjects, using ^123^I-MIBG and ^123^I-FP-CIT SPECT, respectively.

## Materials and methods

### Participants

#### Isolated REM sleep behaviour disorder

Twenty subjects with iRBD (confirmed with video-polysomnography [22]) were evaluated with baseline (age 62.9 ± 5.1 years, 18 males, 2 females) and follow-up ^18^F-FDG PET imaging (age 66.4 ± 5.2 years, interval 3.7 ± 0.6 years; range 2.6–4.6 years), as well as motor, cognitive, and olfactory testing (Sniffin Sticks: identification subscore). Seventeen of these iRBD subjects also underwent ^123^I-FP-CIT SPECT and ^123^I-MIBG SPECT at baseline. All 20 iRBD subjects were recruited in an ongoing longitudinal study at the Philipps-Universität Marburg, Germany (*n* = 17), and the University Medical Center of Groningen (UMCG), The Netherlands (*n* = 3). The details of this study are provided elsewhere [21, 23]. We reported the patients’ clinical status (converted or not converted) at the last available follow-up. Appendix S1 contains the Motor, Cognitive, and Olfactory Assessments.

#### Healthy controls

We included ^18^F-FDG PET scans of 56 healthy controls (HCs) (33 male/23 female, age 63.6 ± 6.2 years) from an existing database at the UMCG. These subjects did not have a history of neurological or psychiatric disease or other chronic illnesses and were not taking psychoactive medication. They had a normal mini mental state examination (MMSE, *N* = 31, 29.5 ± 0.8) or Montreal Cognitive Assessment (MoCA, *N* = 15, 28.4 ± 1.5). Longitudinal data was not available for these controls. This study used their scans as a reference for the voxel-wise single-subject analysis (see “Voxel-wise single-subject analysis”).

All participants provided informed consent. The protocols conformed to the Ethical Standards of the Helsinki Declaration for the protection of human subjects.

### Imaging acquisition and processing

See S2 for details of the acquisition protocol and pre-processing of ^18^F-FDG PET, ^123^I-MIBG, and ^123^I-FP-CIT SPECT images.

### Image analyses

#### Univariate approach: metabolism topography and regional changes over time

##### Voxel-wise single-subject analysis

^18^F-FDG PET processing was based on an optimised semi-quantitative procedure (SPM-based model) [7], validated in clinical research settings for differential diagnosis, including movement disorders [9, 24, 25]. The optimised SPM procedure consists of a statistical comparison between a patient and a database of HC (*n* = 56). Age was entered as a nuisance variable (see S3 for details on the method). This SPM comparison model generates contrast images which are then converted into t-maps and thresholded at *p* < 0.05 (FWE-corrected at the cluster level (*K* > 100 voxels)). An expert rater visually assessed the resulting thresholded SPM t-maps for each individual at both time points. The ratings allowed stratification of each scan according to the previously identified hypometabolism patterns [24]. The contrast images (S3) were entered into two second-level analyses: (i) a voxel-wise analysis and (ii) a region of interest (ROI) analysis. We decided to investigate these hypo- and hypermetabolism values because they represent the significant differences with controls whilst controlling for the ageing effect.

##### Voxel-wise progression analysis

We evaluated the voxel-wise progression of hypo- and hypermetabolism from baseline to follow-up by applying repeated measures ANOVA implemented in SPM12. We used the contrast images already corrected for an age effect from the first-level analysis. *p*-value < 0.005, FWE-corrected at the cluster level, *K* > 100.

##### ROI-based analysis

We extracted mean values in ROIs from the contrast images, using 116 ROIs obtained from the Automated Anatomical Labelling atlas. Regional changes between baseline and follow-up were tested with a one-way ANOVA with repeated measures, Bonferroni corrected for multiple comparisons. The brain regions that showed significant changes over time in the whole group were selected for further analysis. The changes in mean values in these regions were compared between iRBD converters and iRBD non-converters. We used a multivariate generalised linear model (*p* ≤ 0.05, Bonferroni corrected for multiple comparisons).

#### Network (pattern) changes over time: PDRP subject scores

##### Parkinson’s disease–related pattern

A PDRP was previously identified in ^18^F-FDG PET scans of 17 (12 M/5F, age 61.5 ± 7.5 years) HCs and 19 (13 M/6F, age 63.9 ± 7.8) PD patients, in the off-levodopa state [26]. PDRP subject scores were calculated for each scan as described previously [21, 27].

### Statistical analyses

Multivariate generalised linear models were used to compare demographic and clinical variables between iRBD converters and non-converters at baseline and follow-up. The statistical threshold was set at *p* < 0.05, Bonferroni corrected for multiple comparisons.

We also implemented receiver operating characteristic (ROC) and correlation models to evaluate (i) the relationship between regional hypo- and hypermetabolism and phenoconversion and (ii) the relationship between clinical and metabolism changes over time.

#### Relationship between regional hypo- and hypermetabolism at baseline and subsequent phenoconversion

Regions significantly different between converters and non-converters at the group level (at baseline and/or follow-up) were selected. The ROC curve analysis was performed for each region. ROIs were entered as predictive variables and the follow-up clinical diagnosis as the diagnostic reference (converted and non-converted). The optimal cut-point (Youden index) was identified [28].

#### Cognitive, motor, and olfactory correlation with 18F-FDG PET measures

Rates-of-change (Δ/years = [score at follow-up − score at baseline] / years of follow-up) were calculated in cognitive (MoCA scores), motor (UPDRS-III scores), and olfactory measures (Sniffin Sticks identification score). Likewise, we calculated the rate-of-change in PDRP *z*-scores and rates-of-change in metabolism in relevant ROIs (those that showed significant changes from baseline to follow-up in the ROI analysis). Bivariate correlation analyses were performed amongst clinical, cognitive, and ^18^F-FDG PET measures (*p* ≤ 0.05 with Bonferroni correction for multiple comparisons). All variables were normally distributed as assessed by the Shapiro-Wilk test.

All statistical analyses were performed using SPSS version 26.0 and R studio.

## Results

### Clinical and demographic features

Sixteen iRBD patients did not convert during follow-up; they showed no differences between baseline and follow-up in UPDRS-III, MoCA, and olfactory tests. Four iRBD patients converted to PD during follow-up, reflected by an increase in UPDRS-III (1.0 ± 0.8 at baseline, 8.8 ± 2.4 at follow-up; *p* = 0.006) scores. Converted subjects had a greater increase of motor scores at UPDRS-III (∆/year: 1.9 ± 0.5) compared to non-converters (∆/year: − 0.11 ± 0.57; *p* = 0.000). See also Supplementary Figure S1A for the graphical representation.

### Single-subject hypometabolic maps at baseline and follow-up—3 scenarios

**Fig. 1:**
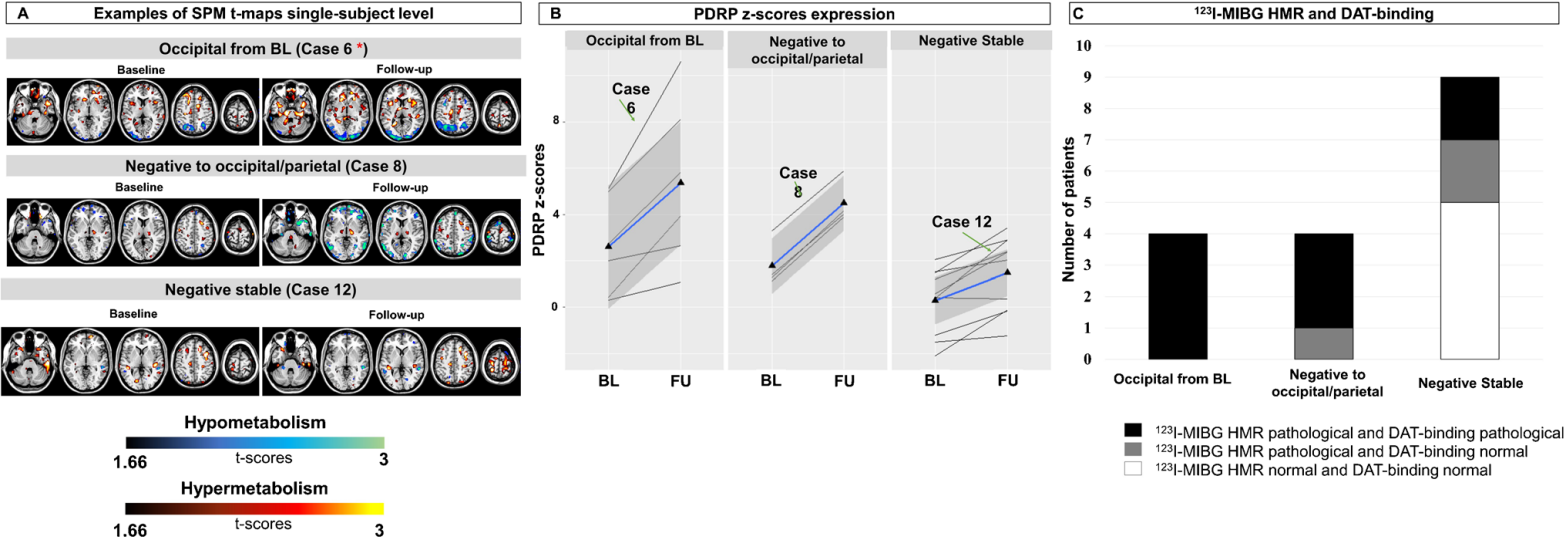
Single-subject hypo- and hypermetabolism maps. Panel **A** shows examples of SPM hypometabolism (black to blue scale) and hypermetabolism (black to yellow scale) t-maps of some iRBD subjects at baseline and follow-up, grouped according to the hypometabolism rating. See supplementary material for the SPM t-maps of all iRBD cases. The red asterisks indicate the iRBD patient converted at follow-up. Panel **B** shows the PDRP *z*-scores of iRBD patients at baseline and follow-up grouped according to the hypometabolism rating. The green arrows highlight the PDRP *z*-scores at baseline and follow-up of three examples of Panel **A** (cases 6, 8, and 12). Panel **C** shows the number of patients with both ^123^I-MIBG HMR and DAT-binding pathological (black), ^123^I-MIBG HMR pathological and DAT-binding normal (grey), and ^123^I-MIBG HMR and DAT-binding normal (white). Abbreviations: SPM, statistical parametric mapping; PDRP, Parkinson’s disease–related pattern; HMR, heart mediastinum ratio of ^123^I-MIBG SPECT images; DAT, dopamine transporter

**Fig. 2:**
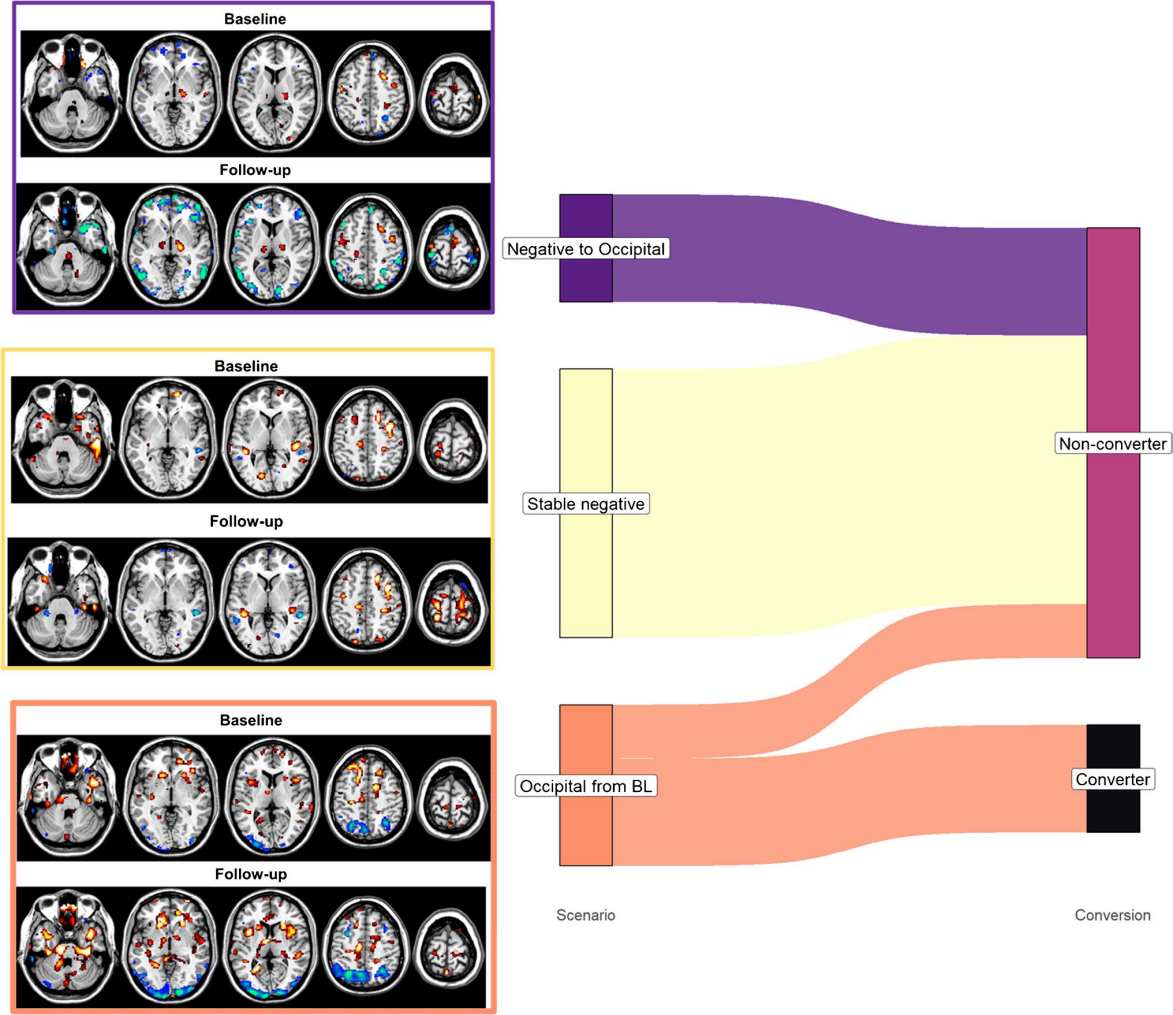
Three scenarios and conversion status. The alluvial plot shows the relationship between three scenarios (first column) and the clinical conversion to PD at follow-up (converter and non-converters) (second column). Only 4 out of 6 patients with occipital hypometabolism from baseline (orange) converted to PD at follow-up (black). On the left side of the alluvial plot are reported examples of the SPM t-maps in each category: negative to occipital (case 8), stable negative (case 12), and occipital from baseline (case 6). Abbreviations: BL, baseline; FU, follow-up

Based on SPM t-map ratings, we identified three possible scenarios: (1) a normal ^18^F-FDG PET scan at both baseline and follow-up (i.e. negative stable over time), (2) a normal scan at baseline but occipito-parietal hypometabolism at follow-up (i.e. from negative to occipito/parietal hypometabolism), and (3) predominant occipital hypometabolism at both baseline and follow-up (i.e. occipital involvement from baseline). See Figs. 1 and 2, and S2–S4.

#### Negative scan, stable over time

Ten out of 20 patients did not show a specific pattern of hypometabolism on the SPM t-maps. Eight of these did not have hypometabolism at all (Figure S2). On both scans, one patient showed hypometabolism in the sensory-motor cortex, and one showed orbitofrontal hypometabolism at baseline and a normal ^18^F-FDG PET scan at follow-up. The hypermetabolism maps of these 10 patients were heterogeneous, ranging from the absence of hypermetabolism to the involvement of the putamen, vermis, cerebellum, sensory-motor cortex, thalamus, and limbic structures. Specifically, at baseline, sensory-motor cortex, vermis, and thalamus were hypermetabolic in half of the patients (i.e. visually detected in 50% of the patients) (Figure S5). At follow-up, sensory-motor cortex (50% of patients), vermis (60% of patients), and parahippocampus/hippocampus (50% of patients) were the most frequent hypermetabolic regions (Figure S5).

These 10 patients had low PDRP *z*-scores at baseline (0.28 ± 1.42) and follow-up (1.48 ± 1.62), which were significantly lower than the iRBD patients with occipital involvement from baseline (*N* = 6) at both time points: baseline (2.59 ± 2.11, stable vs. occipital from baseline *p* = 0.038) and follow-up (5.36 ± 3.56, stable vs. occipital from baseline *p* = 0.013). In nine of them, ^123^I-MIBG SPECT and ^123^I-FP-CIT SPECT were performed. Four (44.44%) had an abnormal ^123^I-MIBG SPECT scan, and 2 (22.22%) had an abnormal DAT-SPECT scan (Fig. 1C).

#### From negative to occipito/parietal hypometabolism

Four patients showed negative ^18^F-FDG PET scans at baseline, but occipital (2 cases) or occipito-parietal (2 cases) involvement at follow-up (Figure S3). In these subjects, we found limited hypermetabolism. At baseline, two patients showed hypermetabolism in the sensory-motor cortex and thalamus (Figure S5). At follow-up, the parahippocampus/hippocampus (75% of patients) were the most frequent hypermetabolic regions (Figure S5). None of these patients converted. The ∆/year PDRP in this group (0.85 ± 0.13) was significantly higher compared to patients with a normal ^18^F-FDG PET scan at both baseline and follow-up (i.e. negative stable over time; *n* = 10; ∆/year PDRP 0.32 ± 0.20; *p* = 0.020). All four (100%) patients in this category had an abnormal ^123^I-MIBG SPECT, and 3 (75%) had abnormal DAT-binding ratios (Fig. 1C).

#### Occipital involvement from baseline

Six patients had occipital hypometabolism at baseline and developed more widespread hypometabolism at follow-up (Figure S4). In 4 out of 6 cases, this also spread to the parietal cortex. These 4 patients converted at follow-up assessment (Fig. 2). The hypermetabolism t-maps of these patients were characterised by the involvement of the cerebellum, sensory-motor cortex, and limbic structures (most prominently, the parahippocampal gyrus). At baseline, half of the patients showed hypermetabolism in the sensory-motor cortex (Figure S5). At follow-up, sensory-motor cortex (67% of patients) and parahippocampus/hippocampus (67% of patients) were the most frequent hypermetabolic regions (Figure S5). Three converted cases develop hypermetabolism in the globus pallidus and one in the thalamus at follow-up (Figure S3, cases 1, 3, 4, and 6). In 3 of these 4 patients ^123^I-MIBG SPECT and ^123^I-FP-CIT SPECT were performed and were both abnormal (100%) (Fig. 1C).

Two of the six patients had a more stable occipital involvement and no relative hypermetabolism. PDRP *z*-scores in these subjects were lower (baseline: mean ± SD: 1.55 ± 1.20; follow-up: 1.86 ± 1.11) than the 4 mentioned previously (i.e. patients converted) (baseline: mean ± SD: 3.30 ± 1.91; follow-up: 6.87 ± 2.56). These two subjects did not convert during follow-up. In one of these two patients, ^123^I-MIBG SPECT and ^123^I-FP-CIT SPECT were performed and were both abnormal.

As a group, the 6 patients with occipital involvement from the baseline measurement had the highest PDRP *z*-scores at baseline and follow-up (baseline: mean ± SD: 2.59 ± 2.11, *p* = 0.038; follow-up: 5.36 ± 3.56) but not the highest rate-of-change per year of PDRP *z*-scores (0.70 ± 0.45).

### Univariate analyses: progressive changes in regional ^18^F-FDG uptake at the group level

#### Voxel-wise approach

**Fig. 3:**
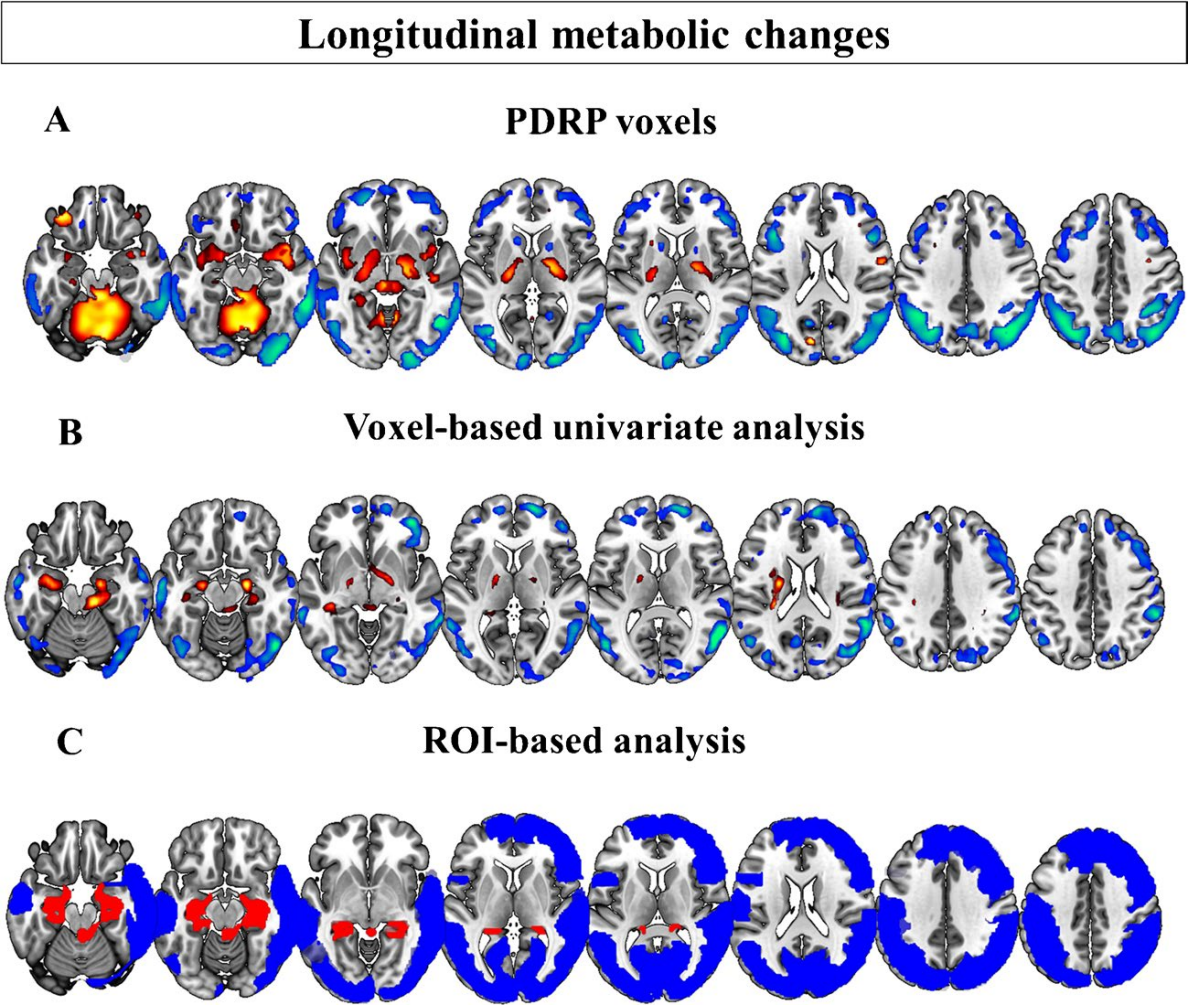
Brain metabolism changes over time in iRBD patients. From top to bottom: **A** Threshold representation of the PDRP (95% confidence interval after bootstrap resampling). The PDRP was previously identified in ^18^F-FDG PET scans from 17 (12 M/5F, age 61.5 ± 7.5 years) HCs and 19 PD patients (13 M/6F, age 63.9 ± 7.8 years, in the off-levodopa state) [26]. The PDRP is consistently characterised by relatively increased activity in the cerebellum, pons, thalamus, putamen/globus pallidus, and motor cortex and relatively decreased metabolism in the lateral premotor and parieto-occipital cortex [10]; **B** voxel-based; and **C** ROI-based hypo- and hypermetabolism changes in our cohort of 20 iRBD patients. Abbreviations: PDRP, Parkinson’s disease–related pattern; ROI, regions of interest

The right middle frontal gyrus, right inferior frontal gyrus (pars opercularis), middle temporal gyrus, bilateral angular and supramarginal gyrus, and middle and inferior occipital gyrus showed progressive hypometabolism. The cerebellum, hippocampus, parahippocampus, and right globus pallidus showed progressive hypermetabolism (Fig. 3B; Table S1).

#### ROI-based approach

Results from the ROI-based approach resembled those of the voxel-based approach. Specifically, the dorsolateral and middle frontal areas, the superior and middle temporal gyrus, and the extended occipito-parietal brain regions showed progressive hypometabolism. The limbic structures (hippocampus and parahippocampus) and cerebellar regions (bilateral vermis and cerebellum) showed a significant increase in hypermetabolism over time (Fig. 3C; Table S2).

The degree of hypometabolism in the occipito-parietal regions (left lingual gyrus, superior, bilateral inferior and middle occipital, and bilateral angular gyrus) was more severe in converters compared to that in non-converters. In addition, hypermetabolism of the bilateral parahippocampus was more pronounced in converters at follow-up than in non-converters (see Table 1).

**Table 1:** Significant differences between converters and non-converters

	Non-converters (*N* = 16)	Converters (*N* = 4)	Partial eta squared	*p*-value-*b*
Baseline—clinical features				
Age, y	63.2 ± 4.6	60.4 ± 7.2	0.051	0.336
Sex (M/F)	14/2	4/0	–	1.000
Age at onset, y	57.0 ± 6.8	55.15 ± 6.9	0.013	0.633
Disease duration, y	6.2 ± 5.3	5.3 ± 4.8	0.005	0.763
UPDRS-I	2.3 ± 1.9	1.3 ± 0.9	0.057	0.312
UPDRS-II	1.4 ± 2.5	0.0 ± 0.0	0.066	0.275
UPDRS-III	2.9 ± 1.9	1.0 ± 0.8	0.158	0.082
UPDRS-total	6.3 ± 5.0	2.25 ± 0.9	0.119	0.137
Sniffin’s test (*I*)	8.4 ± 3.6	4.5 ± 4.1	0.167	0.073
MoCA	26.9 ± 2.0	26.8 ± 2.2	0.001	0.872
PDRP *z*-scores	0.8 ± 1.40	3.3 ± 2.2	0.319	**0.009**
Baseline—hypo/hypermetabolism				
L lingual gyrus^a^	− 0.46 ± 0.49	0.28 ± 0.39	0.301	**0.012**
L occipital superior gyrus^a^	− 0.37 ± 0.58	0.33 ± 0.61	0.204	**0.046**
R occipital superior gyrus^a^	− 0.29 ± 0.47	0.47 ± 0.49	0.317	**0.010**
L occipital middle gyrus^a^	− 0.15 ± 0.58	0.82 ± 0.6	0.333	**0.008**
R occipital middle gyrus^a^	− 0.12 ± 0.49	0.74 ± 0.31	0.376	**0.004**
L occipital inferior gyrus^a^	− 0.16 ± 0.64	0.66 ± 0.15	0.260	**0.022**
R occipital inferior gyrus^a^	− 0.17 ± 0.64	0.52 ± 0.2	0.200	**0.048**
L angular gyrus^a^	− 0.06 ± 0.57	0.96 ± 0.53	0.370	**0.004**
R angular gyrus^a^	0.16 ± 0.67	0.91 ± 0.46	0.200	**0.048**
Follow-up—clinical features				
Age, y	66.8 ± 4.7	64.4 ± 7.2	0.037	0.415
UPDRS-I	2.3 ± 2.1	1.0 ± 2.0	0.066	0.273
UPDRS-II	1.8 ± 1.5	4.0 ± 2.8	0.207	**0.044**
UPDRS-III	2.4 ± 1.9	8.8 ± 2.4	0.650	**0.001**
UPDRS-total	6.7 ± 3.3	13.8 ± 4.6	0.413	**0.002**
Sniffin’s test (I)	7.9 ± 4.5	4.8 ± 4.9	0.077	0.235
MoCA	28.1 ± 1.4	27.00 ± 1.41	0.107	0.159
PDRP *z*-scores	2.3 ± 1.9	7.1 ± 2.9	0.488	**0.001**
Follow-up—hypo/hypermetabolism				
L occipital superior gyrus^a^	0.04 ± 0.57	0.9 ± 0.5	0.295	**0.013**
L occipital middle gyrus^a^	0.14 ± 0.72	1.23 ± 0.77	0.282	**0.016**
R occipital middle gyrus^a^	0.18 ± 0.64	1.46 ± 0.44	0.438	**0.001**
L occipital inferior gyrus^a^	0.18 ± 0.79	1.36 ± 0.5	0.305	**0.012**
R occipital inferior gyrus^a^	0.18 ± 0.82	1.48 ± 0.47	0.338	**0.007**
L angular gyrus^a^	0.25 ± 0.73	1.59 ± 0.73	0.371	**0.004**
R angular gyrus^a^	0.36 ± 0.91	1.64 ± 0.5	0.287	**0.015**
L parahippocampus^b^	0.38 ± 0.43	0.87 ± 0.25	0.210	**0.042**
R parahippocampus^b^	0.34 ± 0.4	0.83 ± 0.25	0.226	**0.034**
Δ/years in hypo/hypermetabolism				
Δ/years R cerebellum 3	0.05 ± 0.06	0.15 ± 0.02	0.370	**0.004**
Δ/years L cerebellum 10	0.03 ± 0.05	0.11 ± 0.04	0.315	**0.010**

General linear model with Bonferroni correction for multiple comparisons

*L*, left; *R*, right; *Δ/years*, rate-of-changes per year; *M*, males; *F*, females; *y*, years; *UPDRS*, Unified Parkinson’s Disease Rating Scale; *MoCA*, Montreal Cognitive Assessment; *PDRP*, Parkinson’s disease–related disorder.

Bold p-values define the significant differences after Bonferroni correction for multiple comparisons (p<0.05).

^a^Hypometabolism mean values extracted from contrast images

^b^Hypermetabolism mean values extracted from contrast images

### Correlation between ^18^F-FDG PET measures and clinical metrics

**Fig. 4:**
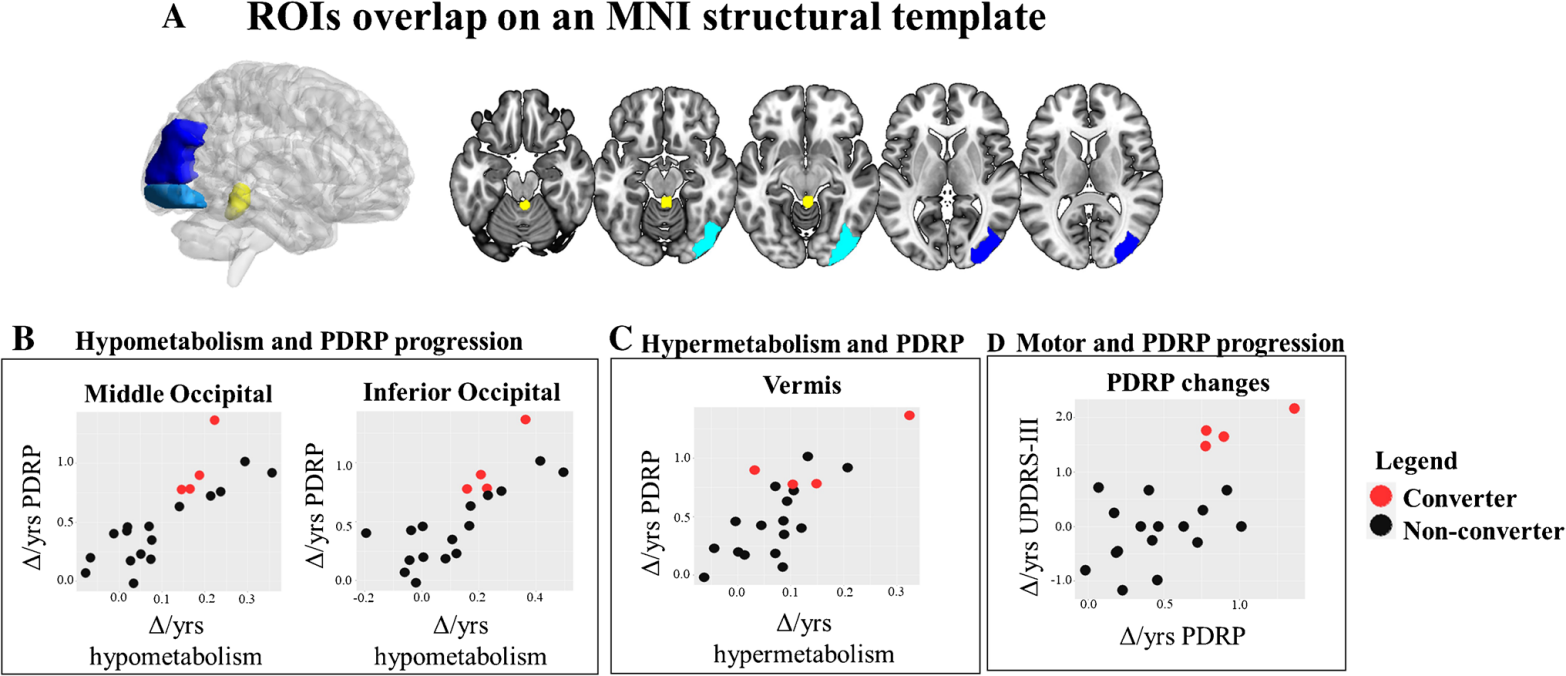
Correlations between PDRP expression and regional metabolic changes over time. The figure shows correlations that survived Bonferroni correction for multiple comparisons. **A** ROIs derived from the AAL atlas that showed significant correlations with PDRP *z*-scores are superimposed on an MNI structural template: right middle (dark blue), inferior occipital gyrus (light blue), and vermis (yellow). **B** Greater rates-of-change in PDRP *z*-scores (Δ/years) correlated with the greater rates of progression of hypometabolism in the middle and inferior occipital gyrus (Δ/years). **C** Likewise, PDRP *z*-scores (Δ/years) were correlated with progression of hypermetabolism in the vermis. **D** Increases in PDRP *z*-scores (Δ/years) correlated with progression of motor symptoms (Δ/years). The four converters (red asterisks) are clearly separated in all scatterplots from the non-converters (black dots). Abbreviations: PDRP, Parkinson’s disease–related pattern

Higher PDRP *z*-scores Δ/years were significantly correlated with the progression of hypermetabolism in the vermis (*r* = 0.77, *p* = 0.014) and progression of hypometabolism in the right inferior occipital (*r* = 0.80, *p* = 0.004) and middle occipital gyrus (*r* = 0.83, *p* = 0.001). Higher PDRP *z*-scores Δ/years also correlated with the progression of motor impairments (higher UPDRS-III Δ/years) (*r* = 0.65, *p* = 0.012) (Fig. 4).

### Relationship between hypo- and hypermetabolism at baseline and subsequent phenoconversion at follow-up

ROC analyses revealed that the right middle occipital gyrus, left inferior occipital gyrus, and left angular gyrus had the highest accuracy for conversion at follow-up: accuracy of 0.922 (95% CI 0.84–0.95), the sensitivity of 1.00, and specificity of 0.875 for the derived Youden index thresholds, 0.36, 0.49, and 0.37, respectively (Table S3).

The presence of occipital hypometabolism at baseline, as assessed by the semi-quantitative rating of single-subject SPM t-maps, showed an accuracy of 0.936 with a sensitivity of 1.00 and specificity of 0.875 (95% CI 0.83–1.00) in identifying iRBD patients who converted to PD at follow-up (Table S3).

## Discussion

This study describes longitudinal brain metabolic changes in a cohort of 20 iRBD patients scanned at baseline and after 4 years. Four patients converted to PD during follow-up. Although most iRBD patients share a final common clinical syndrome, their trajectories towards this common phenotype may differ. We hypothesised that this heterogeneity might be reflected in the changes in brain hypometabolism and hypermetabolism individual maps over time. To understand the individual trajectories of our iRBD patients, we analysed SPM t-maps at the single-subject level, which revealed three scenarios: (1) a normal ^18^F-FDG PET scan at both baseline and follow-up; (2) a normal scan at baseline, with occipital/occipito-parietal hypometabolism at follow-up; and (3) occipital hypometabolism at both baseline and follow-up. Subjects who converted to PD during the study pertained to this third scenario. The presence of occipital hypometabolism on the SPM t-map at baseline gave a 93.6% accuracy (sensitivity: 1.000 and specificity: 0.875) for the prediction of conversion to PD during follow-up. It must be mentioned that these values (accuracy, sensitivity, and specificity) should be interpreted with caution. The high accuracy of occipital hypometabolism in identifying converters might be overestimated because of the sample size; it needs validation in a larger cohort of patients to estimate its effective performance as a predictive biomarker. However, ROC curves at least suggest—in accordance with the single-subject t-maps’ visual rating—an early occipital vulnerability in patients with a short-term conversion (3–4 years) to PD. Occipital hypometabolism is a hallmark feature of DLB [29] and is associated with a faster progression towards dementia in PD [9]. This might suggest that our converters may develop a phenotype with more pronounced cognitive deterioration (PD dementia) as the disease progresses. It is still largely unknown what drives occipital hypofunction [30]. It is probably not related to α-synuclein aggregation in the occipital brain regions [31], but perhaps to an epiphenomenon related to the degeneration of cholinergic projections to the occipital cortex [32–34]. Importantly, our results show that initially negative ^18^F-FDG PET scans can become abnormal in just 4 years. Four of the 20 iRBD subjects originally had a normal scan but developed occipito/occipito-parietal hypometabolism at follow-up. Amongst the iRBD converters, we observed the presence of occipital hypometabolism 3–4 years before conversion to PD (years between baseline and conversion: mean ± SD: 3.63 ± 0.49, range: 3.14–4.16). This suggests that patients who show occipital hypometabolism (at follow-up) are likely to phenoconvert in the upcoming years.

Ten patients (50%) had a normal ^18^F-FDG PET scan at both baseline and follow-up. We speculate that a negative ^18^F-FDG PET scan in iRBD could indicate three possible scenarios: (1) the patient does not have an α-synucleinopathy, (2) the patient has an α-synucleinopathy but are early in the disease course, or (3) the patient will develop PD within a few years but will only have limited cognitive symptoms. The ^123^I-FP-CIT SPECT and ^123^I-MIBG SPECT results support the third scenario: two iRBD patients with a normal ^18^F-FDG PET scan at baseline and follow-up had abnormal ^123^I-FP-CIT and ^123^I-MIBG SPECT at baseline. Pathological values of ^123^I-MIBG SPECT highly indicate the presence of prodromal PD or DLB [35, 36]. Moreover, ^123^I-FP-CIT SPECT abnormalities suggest that a short-term phenoconversion (3–5 years) can be expected [37]. At follow-up, our two patients had a dopaminergic deficit for at least 3–4 years (pathological ^123^I-FP-CIT SPECT already at baseline), thus having a high risk of conversion in the upcoming years. However, contrary to the other patients with abnormal ^123^I-FP-CIT SPECT at baseline (Figures S3 and S4), they did not develop occipital hypometabolism at follow-up (Figure S2). On this basis, we speculate that these iRBD subjects may develop a PD subtype without dementia*.* A previous longitudinal ^18^F-FDG PET study in PD has shown that PD patients who do not deteriorate cognitively during long-term clinical follow-up have either a completely normal ^18^F-FDG PET scan or very limited cortical hypometabolism, for instance, in the sensory-motor cortex [9]. It could be argued that having a normal ^18^F-FDG PET scan has a limited negative predictive power for phenoconversion in the longer term (approximately > 4 years), but this conclusion requires further long-term follow-up. In addition, these findings could also indicate that abnormal ^123^I-FP-CIT and ^123^I-MIBG SPECT reflect earlier events compared to ^18^F-FDG PET (all patients with an abnormal ^18^F-FDG PET scan also had an abnormal ^123^I-FP-CIT and ^123^I-MIBG SPECT), representing sensitive early biomarkers for the underlying pathological process.

Our longitudinal results explain why findings in cross-sectional ^18^F-FDG PET studies are heterogeneous and sometimes conflicting [38]. iRBD is intrinsically heterogeneous: in any cohort, a variable proportion of patients will develop DLB, PD, or MSA at an unknown time interval. In a cross-sectional setting, each individual is scanned at a different time point on his/her disease progression towards manifest PD/DLB (or MSA). This aspect can explain why different cohorts of iRBD patients present variable prevalence of abnormal and normal brain ^18^F-FDG PET scans. In our study, 50% of iRBD showed normal brain ^18^F-FDG PET scans. This differs from what was described in a previous cross-sectional study (only 5 iRBD patients showed normal brain scans) [39]. The two cohorts might have patients in different disease stages and/or clinical trajectories. The prevalence of abnormal scans in our iRBD population might increase over time with the disease progression. On the other hand, a higher percentage of negative scans can also represent a higher percentage of patients converting to a more benign form of PD without cognitive deterioration [9]. Another source of variability may be the presence of compensatory mechanisms. Baril and colleagues [13] found significant regional hypoperfusion in the anterior frontal and lateral parietal-temporal cortex, which disappeared at follow-up. We found a similar trajectory in one of our cases (Figure S2, case 17). This patient showed hypometabolism in the frontal cortex at baseline, which was normalised completely on follow-up imaging. The cause of this phenomenon is unclear. All the above underscores the necessity of longitudinal studies with a large sample and multiple brain scan acquisitions to understand the neurodegenerative process underlying this proteinopathy spectrum.

The SPM t-maps of hypermetabolism showed a high degree of topographical variability. Specifically, hypermetabolism variably involves the cerebellum, sensory-motor cortex, thalamus, putamen, and limbic regions (hippocampus and parahippocampus). Such relative increases have been regarded as artefacts of global mean normalisation [40], but there is also evidence that hypermetabolic areas are important reflections of the pathophysiological process [41, 42]. Findings of hypermetabolism in iRBD have been inconsistent, possibly due to the aforementioned heterogeneity of iRBD cohorts. In one study, hyperperfusion of the hippocampus was also found at baseline and was associated with conversion to PD or DLB within 3 years of follow-up [14]. Kim et al. studied ^18^F-FDG PET scans of 20 iRBD patients at baseline and after 2 and 4 years and found progressive hypermetabolism in the bilateral putamen, cuneus, and lingual gyrus. Hypermetabolism in the bilateral putamen was associated with phenoconversion (3 to PD, 4 to DLB). In our cohort, only 2 out of 6 patients with occipital hypometabolism at baseline did not convert during follow-up. These two patients were also the ones without any significant hypermetabolism at both time points (Figure S4, case 2 and case 5), suggesting that hypermetabolism may also have a role in predicting short-term phenoconversion. Indeed, the four patients who converted at follow-up all showed hypermetabolism in the cerebellum, sensory-motor cortex, and limbic structures and either putamen, thalamus or globus pallidus (Figures S4 and S5).

The results of the voxel-wise and ROI-based analyses mirrored those of the single-subject SPM t-maps. These data-driven analyses also showed a significant progression in hypometabolism at follow-up in frontal, occipital, and parietal regions and hypermetabolism in cerebellar and limbic regions (bilateral hippocampus and parahippocampus). Again, hypometabolism in occipito-parietal regions was significantly more severe in converters compared to non-converters at both baseline and follow-up. The presence of baseline hypometabolism in the right occipital middle gyrus, left inferior occipital gyrus, or right angular gyrus gave the highest accuracy (AUC: 0.922) compared to the other tested ROIs (lingual gyrus, superior occipital gyrus, and parahippocampus) for identifying the converters at follow-up (Table S3). In addition, iRBD converters showed a faster progression of hypermetabolism in the cerebellum and cerebellar vermis.

The brain regions with a significant progression of hypo- or hypermetabolism resemble the topography of the PDRP (Fig. 3). The PDRP is consistently characterised by relatively increased activity in the cerebellum, pons, thalamus, putamen/globus pallidus, and motor cortex and relatively decreased metabolism in the lateral premotor and parieto-occipital cortex [10]. We previously described PDRP expression *z*-scores in this cohort [21]. We showed that PDRP expression increased from baseline to follow-up and that rates-of-change (Δ/years) in UPDRS-III scores were significantly correlated with rates-of-change (Δ/years) in PDRP *z*-scores (Fig. 4). However, in that study, the contribution of single brain regions to PDRP expression in individuals with iRBD was not addressed. In the current study, we show that PDRP *z*-score Δ/years is correlated with the progression of hypometabolism in the occipital cortex and hypermetabolism in the cerebellar vermis (Fig. 4). These results suggest that the increased expression of PDRP in iRBD patients over time is mainly driven by progressive hypometabolism in occipital regions and progressive hypermetabolism in the cerebellum.

This study has some limitations, mainly related to the small sample size and relatively short follow-up. Furthermore, the workup of cognitive changes was limited as we did not perform an extensive neuropsychological evaluation. This limits the assessment of hypometabolic changes in relation to cognitive deterioration and the risk of DLB. Moreover, we do not have repeated measures for age-matched healthy controls for comparison. That said, our results remained significant even after correction for an age effect.

In conclusion, our results suggest that occipital hypometabolism at baseline is a risk factor for short-term conversion from iRBD to manifest PD. Increases in PDRP expression are mainly driven by progressive hypometabolism in this region and progressive cerebellar hypermetabolism. We have shown that repeat scanning is pertinent in this condition and that single-subject SPM t-maps can help map the individual trajectories of patients. Also, our findings support using multiple biomarkers for the stratification of iRBD patients. To further explore the predictive value of ^18^F-FDG PET semi-quantifications and other molecular biomarkers (e.g. ^123^FP-CIT SPECT), a larger, multicentre longitudinal ^18^F-FDG PET study in iRBD is ongoing.

### Supplementary Information

Below is the link to the electronic supplementary material.

Supplementary file1 (DOCX 7961 KB)

## Data Availability

The data that support the findings of this study are available on request from the corresponding author. The data are not publicly available due to privacy or ethical restrictions.
