# Supplementary material for: Occipital hypometabolism is a risk factor for conversion to Parkinson’s disease in isolated REM sleep behaviour disorder
Source: Eur J Nucl Med Mol Imaging. 2023 Jun 13;50(11):3290–301. doi: 10.1007/s00259-023-06289-y (PMC10542098; doi:10.1007/s00259-023-06289-y)
Supplement: Supplementary file 1 — Supplementary file1 (DOCX 7961 KB) [file 259_2023_6289_MOESM1_ESM.docx]

**Supplementary materials**

**S1. Motor, Cognitive, and Olfactory Assessment**

All iRBD patients underwent baseline and follow-up motor, cognitive and olfactory examinations. Specifically, the Unified Parkinson’s Disease Rating Scale, Part III (UPDRS-III; motor examination), was used for motor assessment (Disease, 2003; Goetz et al., 2008), the Montreal Cognitive Assessment (MoCA) for cognitive evaluation (Gagnon et al., 2010), and the Sniffin’ Sticks 16-item olfactory odour identification test (Mahlknecht et al., 2016) for olfaction. A 5-point change in the UPDRS-III was considered to be clinically significant (Shulman et al., 2010). MoCA scores ≤25 out of 30 and Sniffin’ Sticks identification score ≤10 out of 16 were considered abnormal (Gagnon et al., 2010; Mahlknecht et al., 2016).

**S2. [18F]FDG-PET, [123I]MIBG-SPECT and [123I]FP-CIT-SPECT acquisition and pre-processing**

Twenty patients with a diagnosis of iRBD (confirmed by video-polysomnography) underwent baseline and follow-up ^18^F-FDG-PET imaging, with an average of 3.7 ± 0.6 years apart. Fifty-six healthy controls (HCs) also underwent baseline ^18^F-FDG-PET imaging. All baseline and follow-up scans were performed on a Siemens Biograph mCT64 or mCT40 PET/CT camera (Siemens, Munich, Germany) at the University Medical Center of Groningen. Images were reconstructed with OSEM3D (3 iterations, 21 subsets), time-of-flight, point-spread-function, and smoothed with a Gaussian 8-mm full-width-at-half-maximum spatial filter. The matrix size was 256 (corresponding to a voxel size of 2 mm × 3.18 mm × 3.18 mm). Central nervous system depressants and any RBD-related medications (i.e., melatonin or clonazepam) were discontinued in all subjects for at least 24 hours before scanning. All scans were spatially normalised to an ^18^F-FDG-PET template in Montreal Neurological Institute brain space (Della Rosa, 2014) using SPM12 software (Wellcome Centre for Human Neuroimaging, London, UK) implemented in MATLAB (version R2019a; MathWorks, Natick, MA, USA) (Della Rosa, 2014).

17 iRBD also underwent a brain ^123^I-FP-CIT-SPECT 3.9 ± 3.3 months (range 0.4 – 13.5 months) before baseline ^18^F-FDG-PET. 15 patients underwent a ^123^I-MIBG-SPECT exam, 3.9 ± 2.3 months (range 0.6 – 8.6 months) before baseline ^18^F-FDG-PET, another two 10.1 and 15.9 months after the second ^18^F-FDG-PET. The ^123^I-FP-CIT-SPECT and ^123^I-MIBG-SPECT scanning, reconstruction, and analysis protocols have been previously published (Janzen et al., 2022; Meles et al., 2017). DAT binding ratios were calculated in the caudate nucleus and putamen bilaterally, using the occipital cortex for reference (i.e., nonspecific binding). Binding ratios that were 2 or more standard deviations lower than age-matched expected control values were considered abnormal. A detailed explanation of the procedure is explained elsewhere (Meles et al., 2017). We computed the heart-to-mediastinum ratio (HMR) of ^123^I-MIBG-binding on 4-hour planar images, and 1.45 was used as a pathological threshold. A detailed explanation of the procedure is explained elsewhere (Janzen et al., 2022).

**S3. [18F]FDG-PET single-subject: contrast Images**

Normalised and smoothed images were tested for relative whole-brain hypo- and hypermetabolism using a two-sample t-test implemented in SPM12, in which the single image was compared with an ^18^F-FDG-PET database of normal controls (N = 56). Age was entered as a nuisance covariate. This SPM comparison model generates contrast images which are then converted into t-maps and thresholded at p < 0.05 (family-wise error (FWE)-corrected for multiple comparisons at the cluster level (K>100 voxels)). The contrast images obtained previously contain the estimated contrasts (i.e. the linear combination of (beta) parameters) of the considered comparison (i.e. a difference between the patient and HC database). The contrast images derived from the patient < HC contrast contain hypometabolism values, in which a higher positive value indicates more severe hypometabolism in the patient compared to the controls. The contrast images derived from the patient > HC contrast contain hypermetabolism values (a higher value corresponds with more pronounced hypermetabolism in the patient).

**Table S1. Hypometabolism and hypermetabolism Voxel-wise increase (BL<FU)**

| **MNI coordinates** | **Brain regions** |
| --- | --- |
| **Progressive hypometabolism over time** | |
| 44 22 39 | Right Middle Frontal gyrus |
| 19 60 7 | Right Superior Frontal Gyrus |
| 44 48 -6 | Right Frontal Middle Orbital cortex |
| 47 18 37 | Right Inferior gyrus pars. Opercularis |
| -57 -15 -16 | Left Middle Temporal gyrus |
| 60 -3 -22 | Right Middle Temporal gyrus |
| 58 -56 -8 | Right Inferrior temporal gyrus |
| -43 -72 -13 | Left Inferior Occipital Gyurus |
| 41 -71 -13 | Right Inferior Occipital Gyrus |
| -35 -72 -13 | Left Fusiform Gyrus |
| 26 -72 -13 | Right Fusiform Gyrus |
| -32 -79 25 | Left Middle Occipital Gyrus |
| 41 -77 25 | Right Middle Occipital Gyrus |
| -45 -62 37 | Left Angular gyrus |
| -59 -44 25 | Left Supramarginal Gyrus |
| 62 -42 37 | Right Supramarginal Gyrus |
| 62 -7 30 | Right postcentral gyrus |
| **Progressive hypermetabolism over time** | |
| -24 -10 -21 | Left Hippocampus |
| 26 -9 -15 | Right Hippocampus |
| 22 -6 1 | Right Globus Pallidus |
| -29 -7 -27 | Left ParaHippocampus |
| 25 -21 -21 | Right Parahippocampus |
| 15 -30 -20 | Right Cerebellum 3 |
| -19 -41 -41 | Left Cerebellum 10 |
| 18 -38 -41 | Left Cerebellum 10 |

**Table S2. Significant progression of hypometabolism and hypermetabolism ROIs (BL<FU)**

|  | BL | FU | p-value-b | Eta square |
| --- | --- | --- | --- | --- |
| **Progressive Hypometabolism over time** | | | | |
| Precentral gyrus Right | -0.35±0.5 | -0.15±0.62 | 0.010 | 0.299 |
| Frontal Superior gyrus Left | -0.39±0.42 | -0.21±0.55 | 0.049 | 0.189 |
| Frontal Superior gyrus Right | -0.4±0.48 | -0.17±0.58 | 0.024 | 0.024 |
| Frontal Middle gyrus Right | -0.17±0.5 | 0.18±0.57 | 0.005 | 0.350 |
| Frontal Inferior gyrus Opercularis Left | 0.02±0.48 | 0.2±0.52 | 0.032 | 0.219 |
| Frontal Inferior gyrus Opercularis Right | -0.01±0.62 | 0.32±0.59 | 0.004 | 0.365 |
| Frontal Inferior gyrus Triangularis Right | -0.02±0.53 | 0.29±0.59 | 0.010 | 0.301 |
| Frontal Superior Medial cortex Left | -0.13±0.55 | 0.09±0.62 | 0.020 | 0.254 |
| Frontal Superior Medial cortex Right | -0.17±0.56 | 0.08±0.53 | 0.020 | 0.254 |
| Calcarine cortex Left | -0.22±0.74 | 0.11±0.98 | 0.033 | 0.217 |
| Calcarine cortex Right | -0.23±0.83 | 0.14±0.8 | 0.010 | 0.300 |
| Cuneus Left | -0.17±0.64 | 0.23±0.86 | 0.002 | 0.411 |
| Cuneus Right | -0.1±0.71 | 0.35±0.91 | 0.000 | 0.515 |
| Lingual Left | -0.31±0.55 | -0.09±0.75 | 0.023 | 0.242 |
| Lingual Right | -0.25±0.76 | 0±0.84 | 0.018 | 0.260 |
| Occipital Superior gyrus Left | -0.23±0.63 | 0.03±0.82 | 0.007 | 0.322 |
| Occipital Superior gyrus Right | -0.14±0.55 | 0.21±0.65 | 0.000 | 0.567 |
| Occipital Middle gyrus Left | 0.04±0.69 | 0.36±0.84 | 0.001 | 0.474 |
| Occipital Middle gyrus Right | 0.05±0.58 | 0.43±0.8 | 0.000 | 0.488 |
| Occipital Inferior gyrus Left | 0±0.66 | 0.42±0.87 | 0.001 | 0.468 |
| Occipital Inferior gyrus Right | -0.03±0.64 | 0.44±0.92 | 0.002 | 0.409 |
| Postcentral gyrus Left | -0.33±0.44 | -0.2±0.47 | 0.045 | 0.196 |
| Parietal superior gyrus Right | -0.23±0.53 | -0.06±0.66 | 0.022 | 0.248 |
| Parietal inferior gyrus Left | 0.14±0.53 | 0.32±0.48 | 0.015 | 0.275 |
| Parietal inferior gyrus Right | 0.09±0.56 | 0.36±0.66 | 0.001 | 0.477 |
| SupraMarginal Gyrus Left | 0.02±0.31 | 0.25±0.39 | 0.002 | 0.418 |
| SupraMarginal Gyrus Right | -0.07±0.46 | 0.22±0.5 | 0.000 | 0.622 |
| Angular gyrus Left | 0.15±0.69 | 0.52±0.9 | 0.003 | 0.383 |
| Angular gyrus Right | 0.31±0.69 | 0.61±0.98 | 0.024 | 0.241 |
| Temporal Superior gyrus Right | -0.25±0.56 | -0.01±0.57 | 0.014 | 0.276 |
| Superior Temporal Pole Right | -0.2±0.68 | 0.01±0.68 | 0.011 | 0.297 |
| Temporal Middle gyrus Left | -0.05±0.34 | 0.18±0.39 | 0.001 | 0.453 |
| Temporal Middle gyrus Right | -0.17±0.35 | 0.15±0.44 | 0.000 | 0.492 |
| Middle Temporal Pole Right | -0.25±0.56 | -0.1±0.45 | 0.035 | 0.213 |
| Temporal Inferior gyrus Right | -0.15±0.37 | 0.1±0.46 | 0.002 | 0.399 |
| Cerebelum Crus1 Right | -0.54±0.55 | -0.37±0.57 | 0.027 | 0.232 |
| **Progressive hypermetabolism over time** | | | | |
| Hippocampus Left | 0.31±0.39 | 0.58±0.41 | 0.001 | 0.610 |
| Hippocampus Right | 0.35±0.41 | 0.51±0.47 | 0.026 | 0.526 |
| ParaHippocampal Left | 0.28±0.41 | 0.48±0.44 | 0.019 | 0.500 |
| ParaHippocampal Right | 0.29±0.44 | 0.44±0.42 | 0.029 | 0.453 |
| Cerebelum 3 Right | 0.1±0.64 | 0.35±0.69 | 0.000 | 0.110 |
| Cerebelum 10 Left | 0.22±0.49 | 0.4±0.54 | 0.002 | 0.285 |
| Cerebelum 10 Right | 0.25±0.56 | 0.36±0.57 | 0.049 | 0.243 |
| Vermis 1 2 | 0.53±0.85 | 0.73±0.8 | 0.026 | 0.388 |
| Vermis 3 | 0.43±0.84 | 0.73±0.9 | 0.001 | 0.325 |
| Results from repeated-measures ANOVA. BL: baseline, FU: Follow-up, p-value-b: corrected for Bonferroni. | | | | |

**Table S3. ROC curves accuracy**

| **Test Result Variable(s)** | **Area Under Curve** | **p-value** | **Asymptotic 95% Confidence Interval** | | **Threshold**  **(Youden)** | **Specificity** | **Sensitivity** |
| --- | --- | --- | --- | --- | --- | --- | --- |
|  |  |  | **Lower Bound** | **Upper Bound** |  |  |  |
| Occipital hypometabolism* | .938 | .008 | .830 | 1.000 | - | 0.875 | 1.00 |
| Lingual gyrus Left | .906 | .014 | .765 | 1.000 | -0.09 | 0.812 | 1.00 |
| Occipital Superior gyrus Left | .813 | .059 | .612 | 1.000 | -0.27 | 0.625 | 1.00 |
| Occipital Superior gyrus Right | .875 | .023 | .710 | 1.000 | -0.05 | 0.687 | 1.00 |
| Occipital Middle gyrus Right | .922 | .011 | .800 | 1.000 | 0.36 | 0.875 | 1.00 |
| Occipital Inferior gyrus Left | .906 | .014 | .773 | 1.000 | 0.33 | 0.813 | 1.00 |
| Occipital Inferior gyrus Right | .922 | .011 | .796 | 1.000 | 0.49 | 0.875 | 1.00 |
| Occipital Middle gyrus Right | .844 | .038 | .671 | 1.000 | 0.23 | 0.813 | 1.00 |
| Angular gyrus Left | .922 | .011 | .800 | 1.000 | 0.37 | 0.875 | 1.00 |
| Angular gyrus Right | .828 | .047 | .630 | 1.000 | 0.31 | 0.625 | 1.00 |
| ParaHippocampal Left | .688 | .257 | .395 | .980 | 0.64 | 0.937 | 0.50 |
| ParaHippocampal Right | .609 | .508 | .330 | .888 | 0.65 | 0.813 | 0.50 |
| PDRP z-scores baseline | 0.828 | .047 | .555 | 1.000 | 2.39 | 0.94 | 0.75 |
| *Occipital hypometabolism identified by the rating of single-subject SPM t-maps | | | | | | | |


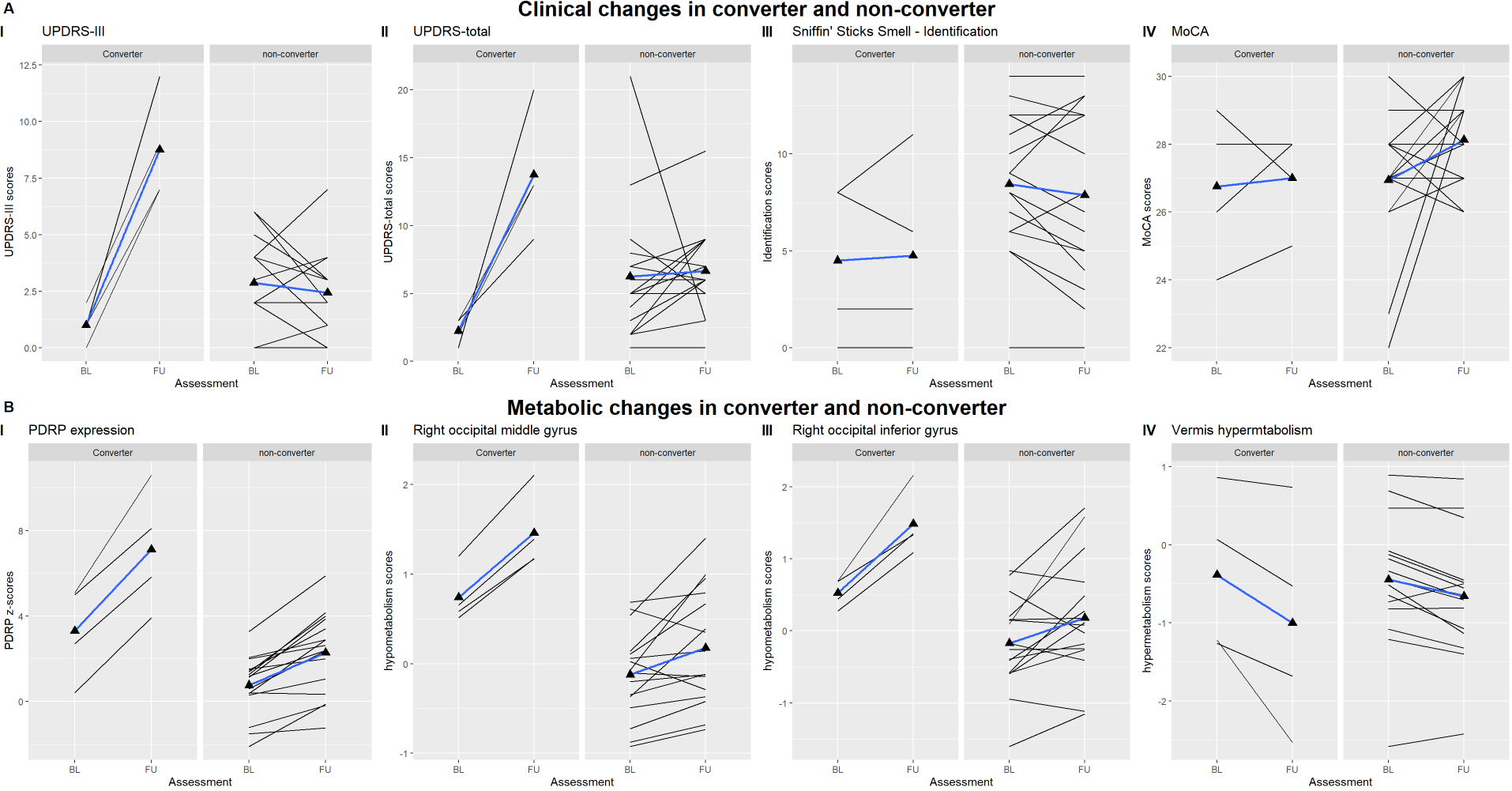


**Figure S1. Clinical and metabolic changes over time in converters and non-converters.** A) Motor (I), clinical (II), olfactory (III) and global cognitive (IV) changes over time in the two groups of iRBD: converters and non-converters. iRBD patients who converted during follow-up are displayed in the left panel; iRBD patients who did not convert during follow-up are displayed in the right panel. Each line represents a single patient’s score, and average scores for each group are indicated with blue lines. The converters had greater changes in UPDRS-III and total scores. No differences emerged in the olfactory and MoCA scores (see Table 1 for the statistics). B) PDRP (I), occipital hypometabolism (II and III), and vermis hypermetabolism (IV) scores over time in the two groups of iRBD: converters and non-converters. Each line represents a single patient’s scores at baseline and follow-up. Average scores for each group are indicated with blue lines. iRBD converted at follow-up showed higher scores at baseline and follow-up in PDRP and occipital brain regions. Also, hypermetabolism in vermis follows the same trend, with a less clear distinction between the two groups (more heterogeneous among patients). Abbreviations: PDRP: Parkinson’s disease-related pattern; BL: baseline; FU: follow-up. UPDRS: Unified Parkinson's Disease Rating Scale, MoCA: Montreal Cognitive Assessment.

**
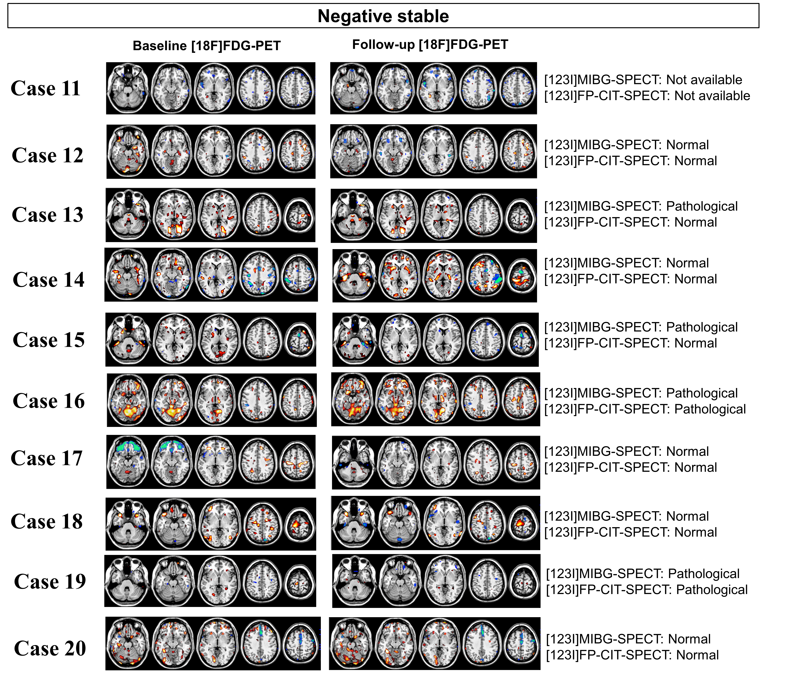
**

**Figure S2.** SPM t-maps of hypometabolism and hypermetabolism in iRBD patients with negative [18F]FDG-PET at baseline and follow-up.

**
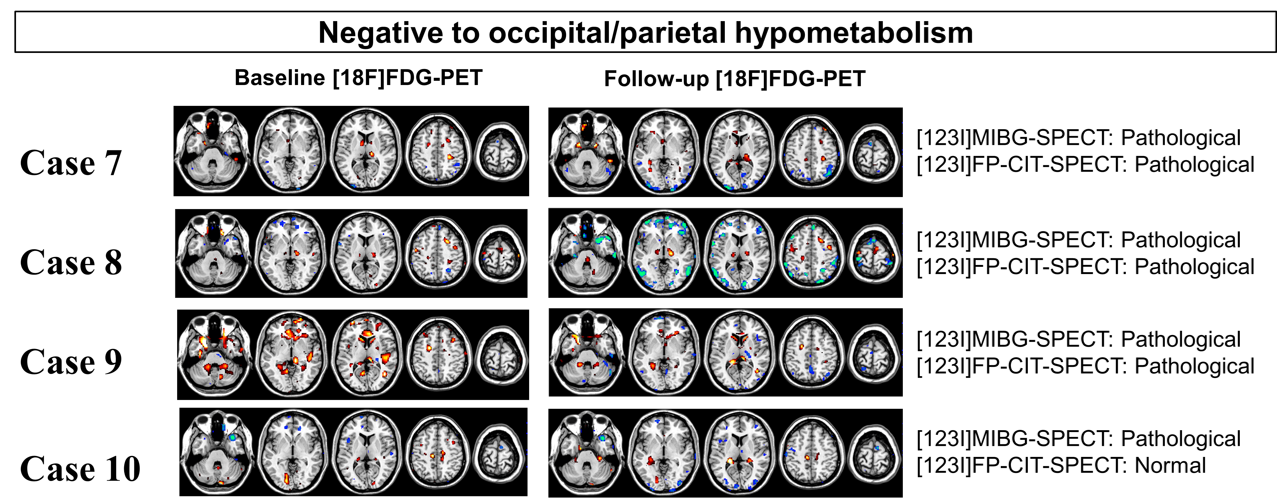
**

**Figure S3.** SPM t-maps of hypometabolism and hypermetabolism in iRBD patients with negative [18F]FDG-PET at baseline and occipital/parietal involvement at follow-up.

**
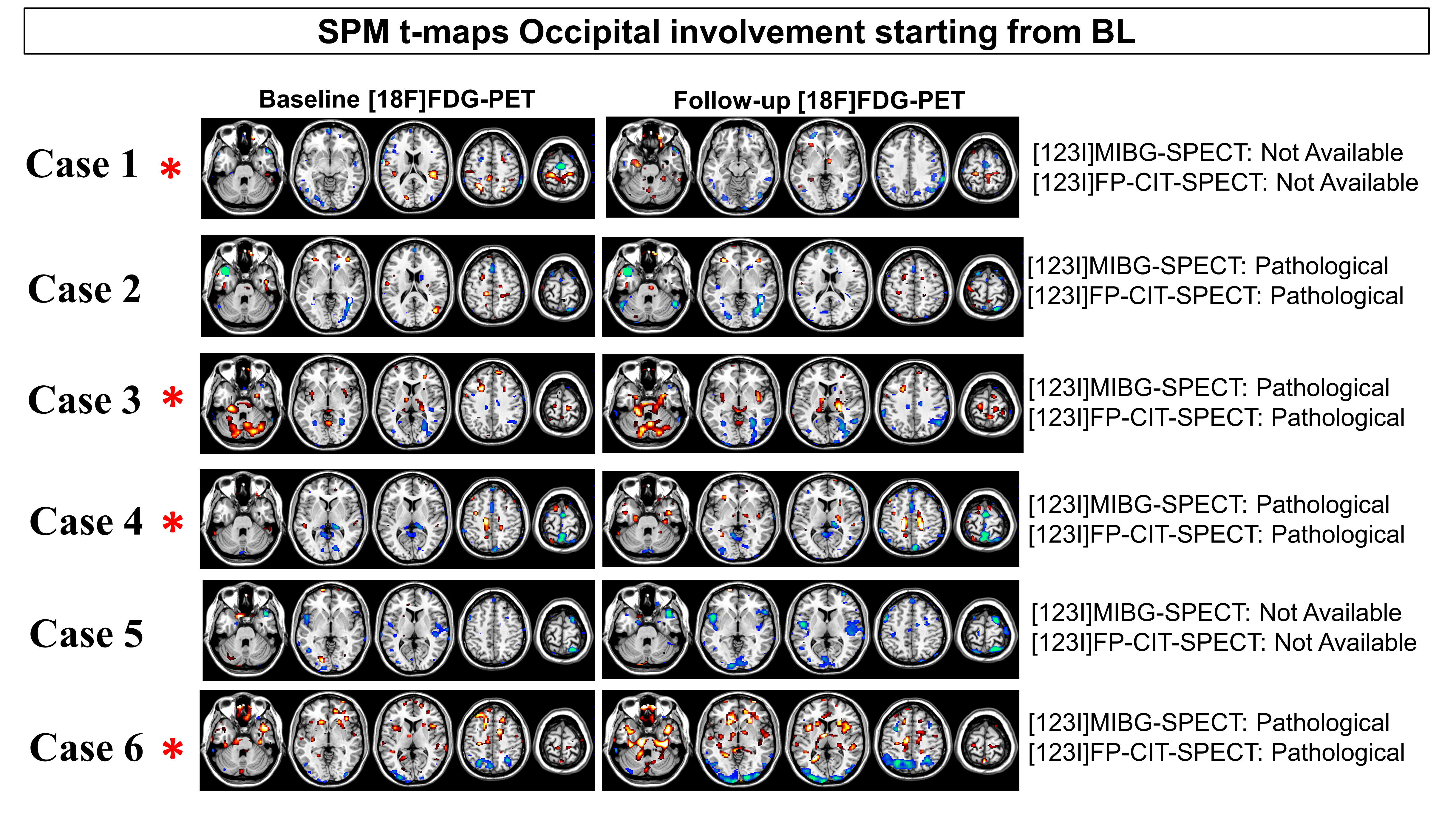
**

**Figure S4.** SPM t-maps of hypometabolism and hypermetabolism in iRBD patients with occipital involvement from the baseline. The red asterisks indicate the iRBD patients converted at follow-up evaluation.

**
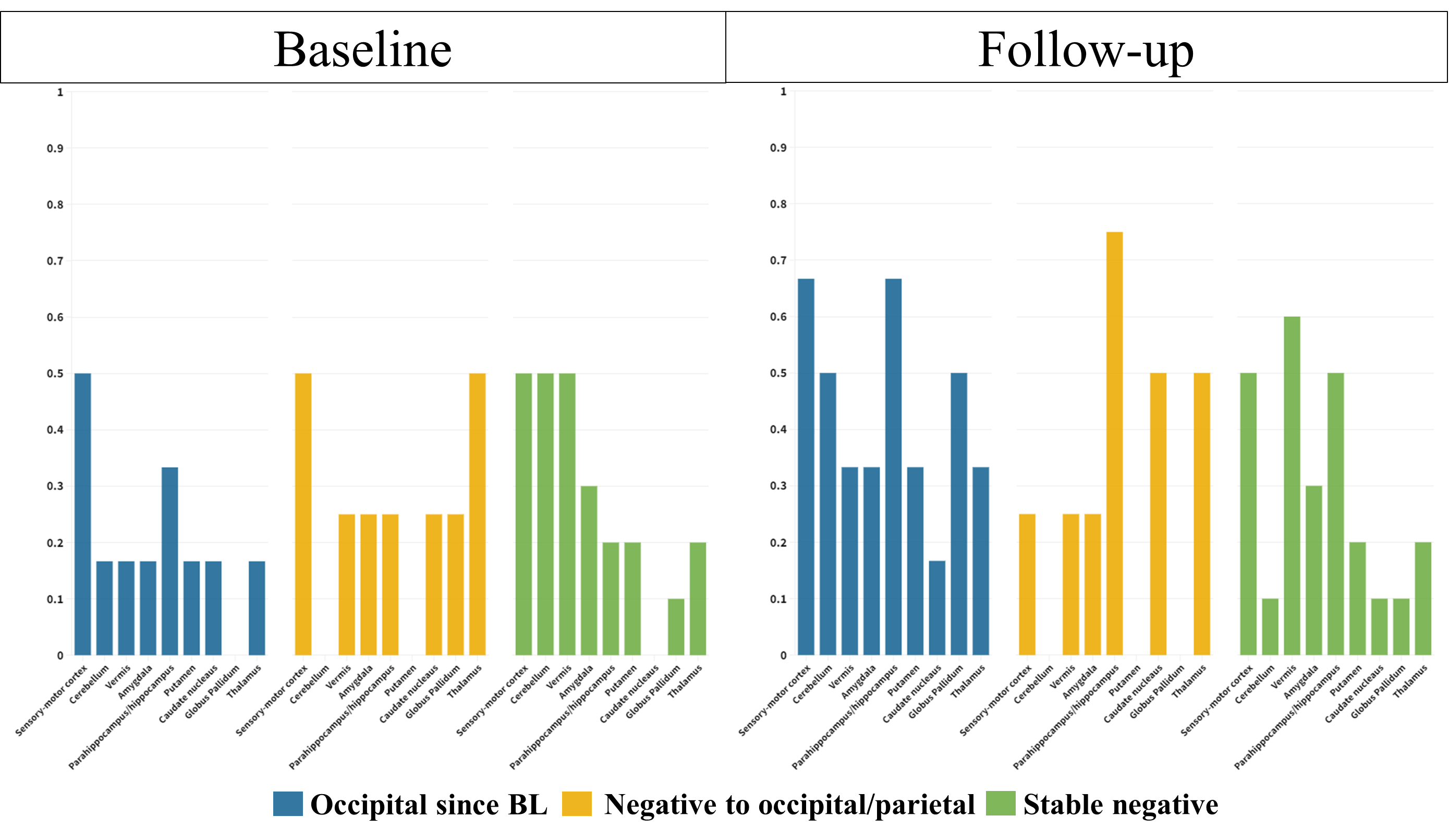
**

**Figure S5. Hypermetabolism percentage distribution among three groups at baseline and follow-up.** The figure represents the percentage of patients showing hypermetabolism in specific regions across the three groups: occipital hypometabolism since BL (blue) (Total number= 6), negative to occipital/parietal hypometabolism (yellow) (Total number = 4) and stable negative (green) (Total number = 10). The baseline is shown in the left panel, and the follow-up in the right one.

**
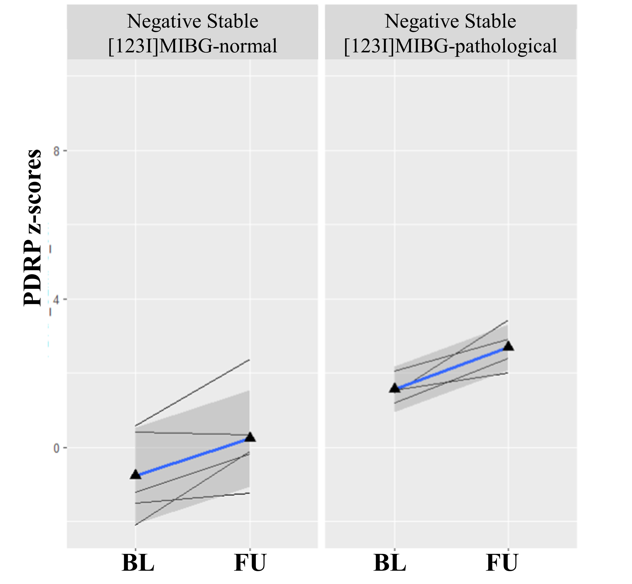
**

**Figure S6. PDRP z-scores at two-time points in [18F]FDG-PET natively stable iRBD patients.** Each line represents a single patient’s PDRP z-scores at baseline and follow-up in [18F]FDG-PET natively stable iRBD patients with [123I]MIBG-SPECT normal (right-panel) or pathological (left-panel). Abbreviations: PDRP: Parkinson’s disease-related pattern; BL: baseline; FU: follow-up.

**Bibliography**

Della Rosa, P. A. (2014). A Standardized [18F]-FDG-PET Template for Spatial Normalization in Statistical Parametric Mapping of Dementia. *Neuroinformatics*, *12*(4), 575–593. https://doi.org/10.1007/s12021-014-9235-4

Disease, M. D. S. T. F. on R. S. for P. (2003). The unified Parkinson’s disease rating scale (UPDRS): status and recommendations. *Movement Disorders*, *18*(7), 738–750.

Gagnon, J., Postuma, R. B., Joncas, S., Desjardins, C., & Latreille, V. (2010). The Montreal Cognitive Assessment: a screening tool for mild cognitive impairment in REM sleep behavior disorder. *Movement Disorders*, *25*(7), 936–940.

Goetz, C. G., Tilley, B. C., Shaftman, S. R., Stebbins, G. T., Fahn, S., Martinez‐Martin, P., Poewe, W., Sampaio, C., Stern, M. B., & Dodel, R. (2008). Movement Disorder Society‐sponsored revision of the Unified Parkinson’s Disease Rating Scale (MDS‐UPDRS): scale presentation and clinimetric testing results. *Movement Disorders: Official Journal of the Movement Disorder Society*, *23*(15), 2129–2170.

Janzen, A., Kogan, R. V, Meles, S. K., Sittig, E., Renken, R. J., Geibl, F. F., Booij, J., Stormezand, G., Luster, M., & Mayer, G. (2022). Rapid Eye Movement Sleep Behavior Disorder: Abnormal Cardiac Image and Progressive Abnormal Metabolic Brain Pattern. *Movement Disorders*, *37*(3), 624–629.

Mahlknecht, P., Pechlaner, R., Boesveldt, S., Volc, D., Pinter, B., Reiter, E., Müller, C., Krismer, F., Berendse, H. W., & van Hilten, J. J. (2016). Optimizing odor identification testing as quick and accurate diagnostic tool for Parkinson’s disease. *Movement Disorders*, *31*(9), 1408–1413.

Meles, S. K., Vadasz, D., Renken, R. J., Sittig‐Wiegand, E., Mayer, G., Depboylu, C., Reetz, K., Overeem, S., Pijpers, A., Reesink, F. E., Leenders, K. L., & Oertel, W. H. (2017). FDG PET, dopamine transporter SPECT, and olfaction: combining biomarkers in REM sleep behavior disorder. *Movement Disorders*, *32*(10), 1482–1486.

Shulman, L. M., Gruber-Baldini, A. L., Anderson, K. E., Fishman, P. S., Reich, S. G., & Weiner, W. J. (2010). The clinically important difference on the unified Parkinson’s disease rating scale. *Archives of Neurology*, *67*(1), 64–70.
